# Society Meetings

**Published:** 1899-10

**Authors:** 


### SOCIETY MEETINGS.

THE American Microscopical Society held its twenty-second annual meeting at Columbus, O., August 17-19, 1899. In point of members present, quality of papers presented and discussed, and good fellowship prevailing among the members, the third Columbus meeting may rank as one of the most successful in the history of the society. Among the papers read especial mention may be made of those by Dr. R. H. Ward, Troy, N. Y., the first president of the society, 1878 to 1880; Professor Simon H. Gage, of Cornell University; Professor H. B. Ward and C. E. Bessey, of the University of Nebraska; C. H. Eigenmann, of the University of Indiana; A. M. Bleile, of the University of Ohio; Dr. A. M. Latham, of Chicago; J. C. Smith, of New Orleans; Dr. A. G. Field, of Des Moines, Ia.; Dr. M. A. Veeder, of Lyons, N. Y., and others.

The presidential address was delivered by Dr. William C. Krauss, of Buffalo, N. Y., on Some medico-legal aspects of diseased cerebral arteries, a portion of which is published on page 159 of the JOURNAL.

The society was handsomely entertained at the residence of Mr. J. F. Stone, on Friday, August 18th, with an imaginary trip to the grand cañons of the Colorado, illustrated with numerous stereopticon views, made, from photographs by Mr. Stone during a visit to that region.

The next meeting of the society will be held conjointly with the American Association for the Advancement of Science, in New York City in 1900. The 1901 meeting will undoubtedly be held in Buffalo.

The officers elected at the Columbus meeting are: President, Prof. A. M. Bleile, of Columbus, O.; vice-president, Dr. M. A. Veeder, of Lyons, N. Y; treasurer, Mr. J. C. Smith, of New Orleans, La.; secretary, Prof Henry B. Ward, of Nincoln, Neb.; executive committee, Dr. J. M. Lamb, Washington, D. C.; Dr. A. T. Kerr, Buffalo, N. Y.

---

THE Medical Association of Central New York will hold its thirty-second annual meeting at Syracuse, N. Y., Tuesday, October 17, 1899, beginning at 10 o'clock in the forenoon. The meetings of this association are second in importance and interest only to the state society and association, and generally attract the active progressive workers of this part of the state. The program is usually a varied and comprehensive one, interesting the general practitioner as well as the specialist. The Syracuse meeting will be an important one, as many changes to the constitution, proposed at the Auburn meeting, will be acted upon. Among such changes the two most important are the changing of the name from Medical Association of Central New York to Medical Association of Central and Western New York, and changing the date of the meeting, third Tuesday in October to the second Tuesday in October, so as not to conflict with the date of the New York State Medical Association.

Any member of a county society, which now includes all Western New York may be elected to permanent membership in the Association by acting as delegate from the county society for two years and the payment of $2.00 initiation fee. Permanent members and delegates attending the meetings are required to pay the annual dues of $1.00.

For the past five years the association has recorded its proceedings in a yearly volume of transactions, each permanent member being entitled to a copy. These volumes have taken a high place in the medical literature of the day and are a credit to the association.

Titles of papers to be read at the Syracuse meeting may be sent to Dr. E. L. Mooney, president, Syracuse, N. Y.; or to Dr. C. A. Vander Beek, Secretary, Rochester N. Y. The present officers of the society are: President, Dr. E. L. Mooney, Syracuse, N. Y.; vice-presidents, Dr. John Gerin, Auburn, N. Y., and A. L. Beahan, Canandaigua, N. Y.; secretary, Charles A. Vander Beek, Rochester; Treasurer, S. L. Elsner, Rochester.

---

THE Wyoming State Medical Society will hold its third annual meeting at Laramie, October 10-11, 1899. The following papers will be read: Medical legislation; its relation to the laity and the medical profession, R. Harvey Reed, Rock Springs; Aortic aneurisms, with report of cases and exhibition of specimens, J. N. Hall, Denver, Colo;. Clinical report of a case of fracture of the nose and upper jaw, E. Stuver, Fort Collins, Colo.; Modern business methods for the medical profession, W. A. Jolly, Rawlins; The administration of anesthetics, E. E. Levers, Almy; The report of some cases in my practice, M. A. Hughes, Salt Lake City, Utah; The disturbances of pregnancy, Charlotte Hawk, Green River; Is high altitude an etiological factor in insanity? C. H. Solier, Evanston; A new method of closing the internal ring in operating for inguinal hernia, Leonard Freeman, Denver, Colo.; Cerebro-spinal

Meningitis, brief report of eleven cases, I. R. Swigart, Laramie; Mountain fever, E. Stuver, Fort Collins, Colo.; The management of abortion with report of cases, John F. Leeper, Casper; The significance of spastic symptoms, Henry W. Coe, Portland, Oregon; Pelvic abscess, P. Hyrup-Pederson, Laramie; Intraligamentous growths, Thomas H. Hawkins, Denver, Col.

---

THE Niagara Falls Academy of Medicine held a regular meeting at the office of Dr. W. A. Scott, 610 Pine Avenue, Monday evening, September 10th. Dr. Scott read a paper on The obstetric forceps.

---

THE Buffalo Academy of Medicine offers the following program during the month of October:

Section on Surgery, Tuesday October 3d, (1) Sulphrenic abscess, Prescott LeBreton; (2) Early diagnosis and treatment of tumors of the breast, L. E. McC. Pomeroy.

Section on Medicine, Tuesday, October 10th, Malaria, Irving P. Lyon: discussion by Charles G. Stockton, Marshall Clinton and John D. Howland.

Section on Pathology, Tuesday, October 17th, Landry's paralysis, Dewitt H. Sherman.

Section on Obstetrics, Tuesday, October 24th, Pus in the pelvic cavity, Herman E. Hayd.

---

THE meetings of the Lake Erie Medical Society and of the Dunkirk and Fredonia Medical Society were held at the Hotel Gratiot, Dunkirk, on September 12, 1899. The following papers were read: Neurasthenia, Dr. Smith, Angola; Surgical knowledge of the prostate, E. J. Meyer, Buffalo; Delirium tremens, Walter H. Vosburg, Dunkirk; Diabetes, Geo. E. Ellis, Dunkirk; Valvular heart disease, H. C. Buswell, Buffalo; Surgery of the hand, MacDonald H. Moore, Fredonia; Some of the uses of electricity in medicine and surgery, Thomas Soles, Westfield.

---

THE Surgical Section of the Buffalo Academy of Medicine held a regular meeting at the Academy Parlors, Tuesday evening, September 5th. The paper of the evening was, The American aborigines from medical and surgical standpoint, by A. L. Benedict.

The Buffalo Academy of Medicine held its quarterly meeting at Alumni Hall, University of Buffalo, Tuesday, September 12th. The

Section of Medicine provided the following program: The medical aspects of crime, Daniel R. Brower, Chicago, Ill.

The Medical Section of the Academy of Medicine held a meeting Tuesday evening, September 19th. Dr. George M. Gould, of Philadelphia, editor of the *Philadelphia Medical Journal*, read a paper on Habits.

---

THE Association of Military Surgeons of the United States held its annual meeting at Kansas City, Mo., September 27-29, 1899. The following order shows the detail of delegates from the National Guard of New York.

GENERAL HEADQUARTERS, STATE OF NEW YORK,
ADJUTANT GENERAL'S OFFICE,
ALBANY, September 7, 1899.

MAJOR ALBERT H. BRIGGS,
Surgeon 65th Regiment, Buffalo, N. Y.

*Dear Sir*—I have the honor to inform you that with the approval of the Governor, the following officers of the National Guard have been nominated as delegates to represent the State of New York at the next meeting of the Association of Military Surgeons of the United States, to be held in Kansas City, Missouri, on September 27, 28 and 29, 1899:

Colonel Nelson H. Henry, Surgeon, National Guard.
Lieut.-Col. Walter E. Lambert, Surgeon 1st Brigade.
Lieut.-Col. George R. Fowler, Surgeon 2d Brigade.
Lieut.-Col. Herman Bendell, Surgeon 3d Brigade.
Lieut.-Col. Floyd S. Crego, Surgeon 4th Brigade.
Major Albert H. Briggs, Surgeon 65th Regiment.
Major Edward T. Marsh, Surgeon 71st Regiment.
Major Bennett S. Beach, Surgeon 22d Regiment.
Major Frederick J. J. Wood, Surgeon 47th Regiment.

These names have been forwarded to Colonel J. D. Griffith, president of the association, with whom you will please communicate.

Respectfully,
AVERY D. ANDREWS, Adjutant General.

---

THE American Association of Obstetricians and Gynecologists held its twelfth annual meeting at Indianapolis, September 19-21, 1899, under the presidency of Dr. Edward J. Ill, of Newark, N. J. It was an enthusiastic and instructive meeting and from every viewpoint must be regarded as a conspicuous success.

The weather was propitious, the attendance was large, the papers excellent, the debates spirited, and the work done was most satisfactory. The committee of arrangements, Dr. L. H. Dunning, chairman, provided most satisfactory for the comfort and convenience of members and visitors and this contributed largely to the general result.

The medical profession of Indiana is strong and was out in large numbers at the sessions. The Medical Association of Marion County, gave a delightful reception at the German House, on Tuesday evening, and the annual dinner was held at the Denison House, cn Wednesday evening, these comprising the social features of the meeting.

Professor W. Japp Sinclair, of Manchester, Eng., attended the meetings and the entertainments and made a fine speech at the dinner. Professor Thaddeus Reamy, of Cincinnati, was also a guest of the Association, participating in all the events. His speech at the dinner was especially a happy effort, and his tribute to the work done by the association was appreciated by all present.

The next meeting will be held at Louisville, September 18-20, 1900. The following-named officers were elected for the ensuing year: President, R. B. Hall, Cincinnati; vice-presidents, L. H. Dunning, Indianapolis, and T. J. Crofford, of Memphis; secretary, William Warren Potter, Buffalo; treasurer, X. O. Werder, Pittsburg; executive council (to fill vacancies), A. Vander Veer, Albany, and L. S. McMurtry, Louisville.

---

The next meeting of the Esculapian Club will be held at the residence of Dr. C. J. Carr, Fillmore Avenue and Genesee Street, Thursday evening, October 19th. The paper of the evening will be read by Dr. F. S. Hoffman.

